# Mediating and Moderating Effects of Internet Use on Urban-Rural Disparities in Health Among Older Adults: Nationally Representative Cross-Sectional Survey in China

**DOI:** 10.2196/45343

**Published:** 2023-09-28

**Authors:** Jing Liu, Junwei Peng, Minyan Chen, Tao Zhang

**Affiliations:** 1 Administrative Office, Yuebei People's Hospital Medical College Shantou University Shaoguan China; 2 Department of Chinese Integrative Medicine Hebei Medical University Shijiazhuang China; 3 Medical Insurance Department Hangzhou Ninth People's Hospital Hangzhou China; 4 Department of Health Policy and Management, School of Public Health Hangzhou Normal University Hangzhou China

**Keywords:** internet use, cognitive function, depressive symptoms, functional disability, mediation analysis, mobile phone

## Abstract

**Background:**

The urban-rural disparities in health outcomes in China are remarkable. The internet has shown the potential to reduce the likelihood of contracting a disease by increasing disease knowledge. However, little is known about the effects of internet use in alleviating health inequities between urban and rural areas.

**Objective:**

This study aimed to examine the mediation and moderation of health disparities between urban and rural older adults through internet use.

**Methods:**

A total of 8223 respondents were selected from the China Health and Retirement Longitudinal Study 2018 data set. Basic activities of daily living, a brief Community Screening Instrument for Dementia, and the Centre for Epidemiologic Studies Depression Scale were used to measure functional disability, cognitive function, and depressive symptoms, respectively. Logistic regressions testing “internet use×urban-rural status” interactions for moderation and Karlson-Holm-Breen decomposition for mediation were performed.

**Results:**

Internet use moderated the urban-rural disparities in cognitive function (odds ratio 7.327, 95% CI 3.011-17.832) and depressive symptoms (odds ratio 1.070, 95% CI 1.037-1.787), but the moderating effects were significant only for those using the internet daily. Karlson-Holm-Breen results showed the suppression effects of using the internet daily (β=.012, 95% CI .002-.021) on the association between urban-rural status and cognitive function. The urban-rural inequality in depressive symptoms was partially attributed to the disparity in internet use (β=−.027, 95% CI −.043 to −.009).

**Conclusions:**

The urban-rural inequalities in mental health are partially attributable to disparities in the prevalence of internet use between the 2 groups. However, using the internet is more beneficial for the psychological health of rural users, thereby alleviating the urban-rural disparities in health. Providing convenient channels for rural older adults to use the internet, improving the ability of rural users to effectively use the internet, and promoting internet popularity in rural areas are effective approaches to reducing urban-rural health inequalities.

## Introduction

### Background

Over the past few decades, China has witnessed rapid economic and social developments. However, the disproportional development between urban and rural areas has plagued the Chinese government and brought unexpected consequences from a public health perspective [1,2]. People residing in rural areas have limited health care services and poor health outcomes compared with their urban counterparts. For example, the rural population was reported to use less hospitalization, presented a higher prevalence of chronic diseases, and had a higher possibility of functional disability and depression [3-6]. Furthermore, the migration of rural young people from rural to urban areas has brought about a massive number of “left-behind” older adults [7,8]. As a result, the disparity between urban and rural health outcomes might be exacerbated in older adults.

The unequal distribution of resources and opportunities (eg, health care services, physical activity facilities, and healthy food) is believed to contribute to the urban-rural inequalities in health [9,10]. In addition, differences in health information and knowledge have received attention. Better accessibility of health information helps individuals increase their knowledge, improve awareness of self-health management, and adopt healthier behavioral patterns [11-13]. However, because of multiple barriers such as geography, distance, and the lack of financial resources and professional physicians, rural residents have limited access to and use of reliable health information [14,15]. A prior study found that rural residents had higher rates of premature morbidity and mortality from diseases such as cancer and heart disease [16], and poor access to knowledge and information on disease prevention is considered to contribute to these rates. In recent years, improving the health literacy of rural residents has become one of the top concerns of the Chinese government.

Encouragingly, the rapid development of the internet in China might provide a promising approach to facilitating rural residents’ access to and use of health information and knowledge. Data have shown that the number of internet users in China has increased to nearly 1 billion, and the popularity rate of the internet reached 70.4% in 2020 [17]. Although most internet users are young and middle-aged adults, the internet has become increasingly popular among older adults with the popularization of smartphones. In 2021, the number of older adults who use the internet will reach 120 million, accounting for 12.2% of the total internet users [18]. Furthermore, the Chinese government launched a series of strategies to promote the development of internet technology in rural areas, such as the “digital village” project. It can be predicted that the increased number of internet users in China will be mainly older adults and rural residents. Considering the association between internet use and health, the popularization of internet in rural areas can be regarded as an important opportunity to reduce health inequities between urban and rural areas.

Previous studies have found multiple effects of internet use on health. Overall, most studies suggest that the effects of the internet on physical and mental health are positive [19-21], although some have found that internet use has either no impact or a negative impact on health outcomes [22,23]. On the basis of the published literature, internet use may affect health outcomes in 2 ways. First, the internet can overcome many social and spatial barriers and provide a convenient way to access health-related information. It helps users improve their knowledge and awareness of self-health management and disease prevention [13,24,25]. Internet users are found to be more likely to have moderate weekly physical activity and eat healthy and are less likely to smoke [26]. In addition, the internet can provide opportunities for residents to interact with health care providers through telemedicine, thereby treating diseases more conveniently [27]. Second, the entertainment attributes of the internet enrich leisure time, reduce loneliness, expand social networks, and strengthen connections with the outside world, thereby reducing the possibility of mental illness [20,28]. Internet users were reported to have more opportunities for social engagement, social activities, and recreation, thus providing individuals who are depressed and lonely with more opportunities for interpersonal and emotional communication [29].

**Figure 1:**
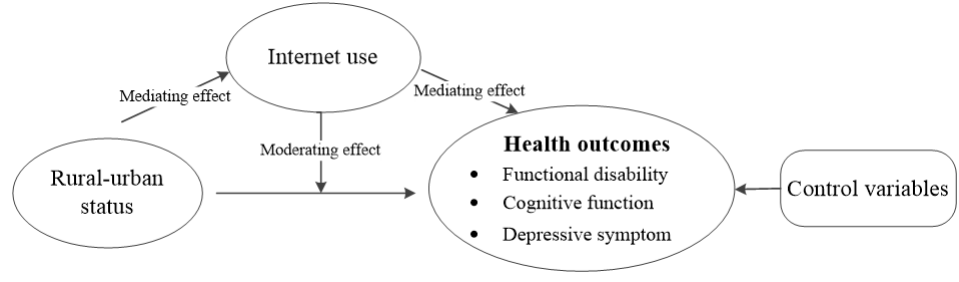
Mediating and moderating models depicting the effect of internet use on the association of urban-rural status with health outcomes.

Although the existing literature has extensively demonstrated the associations between internet use and physical and psychological health, whether these associations vary between urban and rural areas is unknown. On the one hand, fundamental cause theory posits that health inequalities emerge and persist because people who are more advantaged in terms of socioeconomic status are better positioned to avail themselves of health-promoting resources [30]. The internet reflects a new means of social resource allocation. However, the internet is found to form a digital divide between urban and rural areas [14]. This digital gap might further affect the urban-rural inequalities in health. Accordingly, we propose the first hypothesis: internet use mediates the association between urban-rural status and health (Figure 1). On the other hand, the gap narrowing theory proposed by Zhang [31] holds that the expansion of the internet has brought high-quality educational resources to rural students that help narrow the gap in urban-rural education. Similarly, the internet might also offer new opportunities for rural residents to access health services and social interaction, thereby alleviating the negative impact of disadvantaged economic status on health and narrowing the urban-rural gap. In view of this, the second hypothesis is proposed: internet use has a moderating effect on the urban-rural disparities in health (Figure 1).

### Objectives

On the basis of the analysis given in the *Background* section, this study attempts to assess the effects of internet use on the urban-rural disparities in health outcomes among older adults using a nationally representative survey in China. Unlike previous studies that have focused on the direct impact of internet use on health, this study expands the existing literature by investigating the moderating and mediating effects of internet use on the association between urban-rural status and health outcomes. In other words, we not only provide empirical evidence on the differential impact of internet use on the health outcomes of urban and rural residents in China but also uncover the contribution of internet use in explaining the urban-rural inequalities in health using the Karlson-Holm-Breen (KHB) decomposition method. Our findings also provide important policy implications for low- and middle-income countries in terms of understanding and narrowing urban-rural disparities in health.

## Methods

### Data Source

Data used in this study were extracted from the China Health and Retirement Longitudinal Study (CHARLS). CHARLS is an ongoing national longitudinal study on individual demographic and socioeconomic status, health status, and related behavioral information of Chinese residents aged ≥45 years, with subsequent exams every 2 or 3 years [32]. CHARLS adopts a multistage probability-proportional-to-size sampling technique to collect high-quality and representative data. Details of the sampling and data collection methods used in CHARLS can be found elsewhere [32].

Although 4 surveys (2011, 2013, 2015, and 2018) have been completed, the measurements for some variables were not consistent across different CHARLS waves. Cognitive function is an important mental health indicator in our study, and it was assessed using the brief Community Screening Instrument for Dementia (CSI-D) in the 2018 wave; however, this tool was not used in the first 3 rounds of investigation. Considering the consistency of the outcome variables in this study, we used data only from the most recent survey in 2018. The fourth survey consisted of 19,816 individuals who were aged >45 years and their spouses from 12,400 households in 450 village- and 150 county-level units. The average response rate reached 84% (19,816/23,590). Among the initial sample, we extracted 8223 respondents for analysis based on the following criteria: they must (1) be aged ≥60 years and (2) provide complete data for all study variables.

### Measurements

#### Outcome Variables

3D indicators are used to measure the comprehensive health of older adults: functional disability, cognitive function, and depressive symptoms. Functional disability was measured based on basic activities of daily living [33,34]. The study participants were asked about their need for assistance in eating, dressing, indoor mobility, bathing, toileting, and continence. Each item had 3 options, including “no assistance needed” with coding 1, “partial assistance needed” with coding 2, and “full assistance needed” with coding 3. If the participant selected 2 or 3 for any of the above 6 items, they were considered to have functional disability [34].

Cognitive function was assessed using CSI-D. This tool consists of two parts: (1) an interview with the study participants to measure their cognitive function and (2) an interview with a close relative to gather information on daily functioning and cognitive decline. The first part consists of 7 cognitive test items, and the summed score ranges from 0 to 9 [35,36]. Generally, a total score of ≤4 indicates severe dementia, and a total score of ≥7 indicates no risk of dementia. The second part of the questionnaire assesses the cognitive performance of those who score 5 or 6. The second part consists of 6 questions assessing changes in the respondents’ daily lives, with a summed score ranging from 0 to 6. Those with a score of 0 or 1 are identified as having dementia [35]. More details on the CSI-D measure can be found in Multimedia Appendix 1.

Depressive symptoms were identified using the 10-item Centre for Epidemiologic Studies Depression Scale. Participants were asked about the frequency of 8 negative effects and 2 positive effects. A 4-point rating was adopted: 0=rarely or none of the time, <1 day; 1=some of the time, 1-2 days; 2=much or a moderate amount of the time, 3-4 days; or 3=most or all the time, 5-7 days. The sum of the 10-item Centre for Epidemiologic Studies Depression Scale scores ranged from 0 to 30, and a score of 10 was used as the cutoff point to identify those who had depressive symptoms [37].

#### Independent Variables

Our primary independent variable of interest is the urban-rural residence status. In the CHARLS questionnaire, the respondents were asked to report their current residential areas.

#### Mediators and Moderators

Internet use was elicited by asking the respondents to answer the question, “Have you used the Internet in the last month.” If yes, they were asked the frequency of internet use in the last month. The rating in terms of frequency ranged from “not regular” (2), “weekly” (3), and “daily” (4). Those who answered no to the first question were assigned 1, indicating “never.”

#### Control Variables

Control variables included demographic characteristics, socioeconomic status, and health care services. Demographic characteristics included age, gender, and marital status. Socioeconomic status included literacy, employment status, wage, pension, subsidy, and pension insurance. Health care–related variables included medical insurance and accessibility to physical examination services. Details of the definition of these measurements are provided in Multimedia Appendix 1.

### Statistical Analysis

First, descriptive statistics were used to analyze the characteristics of urban and rural respondents, and chi-square and 2-tailed *t* tests were used to examine the differences between urban and rural internet use. Second, binary logistic regressions were established to assess urban-rural disparities in health outcomes by controlling for covariates.

The moderating effect of internet use was assessed by establishing separate logistic regressions, including an interaction term between the moderator of interest and urban-rural status, while controlling for all covariates described in the models. If the interaction term was statistically significant at *P*<.05, it indicated that internet use was the moderator [38,39].

Mediation analysis was conducted using the KHB decomposition method. This method allows for decomposing the total effect of urban-rural status on health into its direct and indirect components. Specifically, it quantifies the change in the association between the independent variable (urban-rural status) and the outcome variables (health outcomes) before and after controlling for mediators (internet use) [40]. These changes are the indirect effects of the mediators. If the indirect effect is significant, it indicates that the effect of independent variables on the outcome variables is partially explained by mediators [41]. In this analysis, it means that part of the effect of urban-rural status is mediated by internet use.

All statistical analyses were performed using STATA (version 16.0; StataCorp), and *P*<.05 was regarded as statistically significant.

### Ethical Considerations

The CHARLS survey was approved by the Ethical Review Committee of Peking University (approval IRB00001052–11015). The survey was anonymous, and the answers were protected by privacy law. Each participant provided written informed consent to participate in this study. There was no requirement for additional ethical approval from the approved data users.

## Results

### Descriptive Results for the Study Variables

Table 1 shows the differences in health outcomes and other characteristics between the urban and rural residents. Surface-level comparisons found that urban older adults performed better in terms of functional disability, cognitive function, and depressive symptoms than their rural counterparts (*P*<.001). In addition, significant differences were observed between urban and rural areas in terms of internet use (*P*<.001). Regarding control variables, urban and rural older adults differed significantly in terms of socioeconomic status and health care services. For example, the proportion of urban older adults covered by pension insurance was higher than that of rural areas (*P*<.001). More older adults in rural areas were still working than those in urban areas (*P*<.001).

**Table 1:** Descriptive statistics of the study variables.

Study variable		Rural (n=5868)	Urban (n=2355)	*P* value	
**Functional disability, n (%)**					<.001
	Yes	2794 (47.61)	996 (42.29)		
	No	3074 (52.39)	1359 (57.71)		
**Cognitive function, n (%)**					<.001
	Yes	1160 (19.77)	165 (7.01)		
	No	4708 (80.23)	2190 (92.99)		
**Depressive symptom, n (%)**					<.001
	Yes	2365 (40.3)	689 (29.26)		
	No	3503 (59.7)	1666 (70.74)		
**Internet use, n (%)**					<.001
	Yes	179 (3.05)	446 (18.94)		
	No	5689 (96.95)	1909 (81.06)		
**Frequency of internet use, n (%)**					<.001
	Never	5689 (96.95)	1909 (81.06)		
	Not regular	16 (0.27)	22 (0.93)		
	Weekly	20 (0.34)	39 (1.66)		
	Daily	143 (2.44)	385 (16.34)		
**Gender, n (%)**					.004
	Male	3083 (52.54)	1154 (49)		
	Female	2785 (47.46)	1201 (51)		
Age (years), mean (SD)		68.07 (6.1)	68.33 (6.4)	.09	
**Marital status, n (%)**					.34
	Married	4812 (82)	1910 (81.1)		
	Others	1056 (18)	445 (18.9)		
**Literacy, n (%)**					<.001
	Yes	2512 (42.81)	1711 (72.65)		
	No	3356 (57.19)	644 (27.35)		
**Wage, pension, or subsidy, n (%)**					<.001
	Yes	4747 (80.9)	2099 (89.13)		
	No	1121 (19.1)	256 (10.87)		
**Employment status, n (%)**					<.001
	Yes	3927 (66.92)	541 (22.97)		
	No	1941 (33.08)	1814 (77.03)		
**Pension insurance, n (%)**					<.001
	Yes	467 (7.96)	1429 (60.68)		
	No	5401 (92.04)	926 (39.32)		
**Medical insurance, n (%)**					<.001
	Yes	5691 (96.98)	2322 (98.6)		
	No	177 (3.02)	33 (1.4)		
**Access to physical examination service, n (%)**					<.001
	Yes	3184 (54.26)	1512 (64.2)		
	No	2684 (45.74)	843 (35.8)		

### Urban-Rural Disparities in Health Outcomes

Table 2 reports the results for 3 logistic regressions using each health outcome as the dependent variable. After adjusting for control variables, we found that older adults residing in urban regions had a lower possibility of functional disability (odds ratio [OR] 0.829, 95% CI 0.723-0.938), cognitive impairment (OR 0.567, 95% CI 0.463-0.693), and depressive symptoms (OR 0.783, 95% CI 0.686-0.893) compared with their rural counterparts.

**Table 2:** Multivariate logistic regression of health outcomes on urban-rural residence status.

Variables (references)		Functional disability, OR^a^ (95% CI)	Cognitive function*,* OR (95% CI)	Depressive symptoms, OR (95% CI)
**Residency (rural)**				
	Urban	0.829^b^ (0.723-0.938)	0.567^c^ (0.463-0693)	0.783^c^ (0.686-0.893)
**Gender (male)**				
	Female	0.693^c^ (0.630-0.762)	1.167^d^ (1.020-1.336)	1.506^c^ (1.364-1.663)
Age		1.992^d^ (1.984-2.000)	1.056^c^ (1.045-1.067)	1.001 (0.993-1.009)
**Marital status (married)**				
	Others	1.173^b^ (1.040-1.322)	1.186^d^ (1.014-1.386)	1.242^b^ (1.099-1.404)
**Literacy (no)**				
	Yes	0.989 (0.896-1.092)	0.308^c^ (0.264-0.358)	0.763^c^ (0.689-0.846)
**Wage, pension, or subsidy (no)**				
	Yes	1.034 (0.917-1.166)	0.909 (0.777-1.063)	0.890 (0.788-1.006)
**Employment status (no)**				
	Yes	0.963 (0.869-1.066)	0.912 (0.793-1.050)	0.844^b^ (0.759-0.939)
**Pension insurance (no)**				
	Yes	0.940 (0.822-1.076)	0.389^c^ (0.289-0.509)	0.615^c^ (0.530-0.713)
**Medical insurance (no)**				
	Yes	0.917 (0.695-1.211)	0.801 (0.578-1.111)	0.887 (0.669-1.177)
**Access to physical examination service (no)**				
	Yes	1.023 (0.935-1.119)	0.729^c^ (0.642-0.827)	0.888^d^ (0.808-0.976)

^a^OR: odds ratio.

^b^*P*<.01.

^c^*P*<.001.

^d^*P*<.05.

### Moderation Analyses

Table 3 presents the effects of internet use on the association of urban-rural status with older adults’ health (Multimedia Appendix 2 provides complete results, including coefficients of the control variables). The results showed that the effects of the interaction between internet use and urban-rural status on cognitive function (OR 7.327, 95% CI3.011-17.832) and depressive symptoms (OR 1.070, 95% CI 1.037-1.787) were significant, but not significant for functional disability, indicating a negative moderation effect of internet use on urban-rural inequalities in these 2 health outcomes. Regarding the frequency of internet use, the slope of the negative association between urban-rural status and cognitive function (OR 8.580, 95% CI 3.018-24.392) and depressive symptoms (OR 1.190, 95% CI 1.037-1.923) was remarkably moderated by using the internet daily.

With regard to those with significant interaction terms in Table 3, we further examined whether the effect of urban-rural status on health varies by internet use (Table 4). The results showed that the impact of urban-rural status on cognitive function was relatively lower among internet users (OR 0.108, 95% CI 0.089-0.402) and those who use the internet daily (OR 0.344, 95% CI 0.003-0.825). In addition, the effect of urban-rural status on depressive symptoms appeared to be not significant among internet users and those who use the internet daily, but its effect was still remarkable for individuals who did not use the internet (OR 0.791, 95% CI 0.690-0.970) and did not use the internet daily (OR 0.787, 95% CI 0.687-0.901).

**Table 3:** Modifying effects of internet use on the association of urban-rural status with the older adults’ health.

				Functional disability, OR^a^ (95% CI)		Cognitive function, OR (95% CI)		Depressive symptoms, OR (95% CI)
**Moderator: internet use**								
	**Urban-rural status (reference=rural)**							
		Urban	0.826^b^ (0.727-0.939)		0.473^c^ (0.380-0.588)		0.802^c^ (0.701- 0.918)	
	**Internet use (reference=no)**							
		Yes	1.491 (0.794-2.799)		0.052^c^ (0.011-0.260)		0.620^d^ (0.299-0.887)	
	Urban-rural status×internet use		0.870 (0.606- 1.248)		7.327^c^ (3.011-17.832)		1.070^d^ (1.037-1.787)	
**Moderator: frequency of internet use**								
	**Urban-rural status (reference=rural)**							
		Urban	0.827^c^ (0.728-0.940)		0.471^c^ (0.378-0.587)		0.799^c^ (0.698- 0.914)	
	**Frequency of internet use (reference=never)**							
		Not regular	1.168 (0.262-5.207)		5.545 (0.668-46.023)		2.321 (0.480- 11.225)	
		Weekly	4.040 (0.383-42.611)		0.515 (0.029-9.102)		1.618 (0.158- 16.587)	
		Daily	1.409 (0.696-2.854)		0.037^b^ (0.005-0.249)		0.515^b^ (0.223- 0.919)	
	Urban-rural status×not regular		0.951 (0.346- 2.613)		0.573 (0.105-3.122)		0.631 (0.210-1.900)	
	Urban-rural status×weekly		0.421 (0.112- 1.591)		4.061 (0.762-21.661)		0.904 (0.241-3.401)	
	Urban-rural status×daily		0.911 (0.610- 1.360)		8.580^c^ (3.018-24.392)		1.190^b^ (1.037-1.923)	

^a^OR: odds ratio.

^b^*P*<.01.

^c^*P*<.001.

^d^*P*<.05.

**Table 4:** Heterogeneity effect of urban-rural status on health by internet use.

Variables and stratification variables				Cognitive function, OR^a^ (95% CI)		Depressive symptoms, OR (95% CI)
**Urban-rural status (reference=rural)**						
	**Internet use**					
		No (n=7589)	0.488^b^ (0.394-0.606)		0.791^c^ (0.690-0.907)	
		Yes (n=625)	0.108^d^ (0.089-0.402)		0.940 (0.541-1.634)	
	**Daily internet use**					
		No (n=7695)	0.494^b^ (0.399-0.611)		0.787^c^ (0.687-0.901)	
		Yes (n=528)	0.344^b^ (0.003-0.825)		1.164 (0.614-2.210)	
**Controls**						
	Yes^e^			N/A^f^		N/A

^a^OR: odds ratio.

^b^*P*<.001.

^c^*P*<.01.

^d^*P*<.05.

^e^Yes represents the control variables being placed in the model as covariates.

^f^N/A: not applicable.

### Mediation Analyses

Table 5 reports the mediation effect of internet use using KHB decomposition. In terms of functional disability, the indirect effect was not significant, indicating that the effect of urban-rural status on functional disability is not mediated by internet use. The urban-rural difference in cognitive function was mediated by using the internet daily (β=.012, 95% CI .002-.021). However, we found that this indirect effect coefficient was opposite to the total and direct effects, which means that the urban-rural disparity in cognitive function is widened after controlling internet use.

In addition, the KHB results also showed that the effect of urban-rural status on depressive symptoms was mediated by internet use (β=−.027, 95% CI −.043 to −.009). Regarding the effects of different frequencies of internet use, only using the internet daily (β=−.024, 95% CI −.039 to −.008) mediated the urban-rural difference in depressive symptoms. This indicates that the urban-rural inequality in depressive symptoms is partially attributable to disparities in internet use.

**Table 5:** Mediation of the relationship between older adults’ health and urban-rural status by internet use.

			Functional disability, β (95% CI)		Cognitive function, β (95% CI)		Depressive symptom, β (95% CI)
**Mediation by internet use**							
	Total effect	−.189^a^ (−.313 to −.064)		−.628^b^ (−.835 to −.421)		−.243^b^ (−.357 to −.111)	
	Direct effect	−.201^a^ (−.325 to −.076)		−.644^b^ (−.852 to −.436)		−.216^b^ (−.349 to −.084)	
	Indirect effect	.012 (−.001 to .025)		.015 (−.012 to .043)		−.027^b^ (−.043 to −.009)	
**Mediation by frequency of internet use (not regular)**							
	Total effect	−.188^b^ (−.312 to −.064)		−.635^b^ (−.842 to −.428)		−.239^b^ (−.371 to −.107)	
	Direct effect	−.189^b^ (−.312 to −.064)		−.637^b^ (−.844 to −.430)		−.240^b^ (−.372 to −.108)	
	Indirect effect	.001 (−.001 to .002)		.002 (−.005 to .008)		.001 (−.001 to .002)	
**Mediation by frequency of internet use (weekly)**							
	Total effect	−.189^a^ (−.313 to −.064)		−.629^b^ (−.836 to −.421)		−.239^b^ (−.371 to −.107)	
	Direct effect	−.188^a^ (−.312 to −.064)		−.642^b^ (−.850 to −.434)		−.242^b^ (−.374 to −.110)	
	Indirect effect	−.001 (−.005 to .003)		.013 (−.013 to .040)		.002 (−.002 to .007)	
**Mediation by frequency of internet use (daily)**							
	Total effect	−.189^a^ (−.313 to −.065)		−.633^b^ (−.840 to .425)		−.242^b^ (−.374 to −.111)	
	Direct effect	−.201^a^ (−.326 to −.076)		−.645^b^ (−.852 to −.437)		−.218^b^ (−.351 to −.086)	
	Indirect effect	.012 (−.001 to .025)		.012^c^ (.002 to .021)		−.024^a^ (−.039 to −.008)	

^a^*P*<.01.

^b^*P*<.001.

^c^*P*<.05.

## Discussion

### Principal Findings

This study examined the effects of internet use on the association between urban and rural status and health outcomes among older adults in China. The results showed that the association between urban-rural status and 2 mental health measures (cognitive function and depressive symptoms) was moderated and mediated by internet use. It sheds some light on how urban-rural disparities in health outcomes in China can be narrowed.

Consistent with previous studies [4,5,42], the regression models showed a greater urban-rural disparity in health for each of the 3 health measures, even after adjusting for demographic characteristics, socioeconomic status, and health care–related factors. In addition to the factors mentioned in *Background* section, an urban-rural dual system in China also causes differences in other health risk factors. For example, urban residents adopt healthier lifestyles and behaviors than their rural counterparts [43]. Recently, bridging the gap between urban and rural areas has become one of the vital tasks of the Chinese government.

This study found that the effects of internet use on urban-rural disparity in functional disability were not significant (*P*=.45). We considered that most older adults had a disability before 60 years of age or were exposed to risk factors for disability at a younger age [44]. Although the internet can provide health-related information and knowledge to prevent disabilities, its effects are not evident for older adults who have been disabled, especially in rural areas. Older adults residing in rural areas, even at retirement age, must engage in agricultural work to earn a living because of a lack of pension insurance [45]. This highlights the importance of providing material assistance to these populations.

More importantly, our results highlighted the negative moderating effect of internet use on the association of urban-rural status with the 2 mental health outcomes. In other words, rural elders benefit more from internet use than their urban counterparts do. There are 2 possible explanations for this finding. First, the acceleration of urbanization in China has led to an increasing number of left-behind older adults and empty nesters in rural areas. They live far from their children and have few opportunities to communicate face-to-face with their children [7,8]. In this case, smartphones provide an important communication tool to connect with their families, thereby helping reduce loneliness and depressive inclination [46]. Second, the internet has various entertainment functions, and it can provide many interesting games and social platforms (eg, Tik Tok and WeChat) for older adults in rural areas. For urban older adults, using the internet is one of the various means of entertainment because they have more access to engaging in different activities, such as square dances, movie theaters, and volunteer activities [47,48]. Correspondingly, the effect of internet use on improving mental health in urban areas was not as significant as that in rural areas. Thus, promoting internet popularization in rural areas is considered an effective approach to reduce the urban-rural inequalities in mental health.

Another notable finding is that the effect of urban-rural status on mental health is partially mediated by internet use. In terms of cognitive function, using the internet daily created a suppression effect. Difference in cognitive impairment between those in urban and rural areas further expands after controlling for internet use. One possible reason is the lower share of rural respondents using the internet or the higher impact of internet use on cognitive performance in the rural group. In addition, the KHB model also found that part of the gap in depressive symptoms between those in urban and rural settings can be attributed to internet use. Previous studies have demonstrated the positive effect of internet use on the reduction of the likelihood of depression [20,28]. However, the poor network infrastructure in rural areas of China restricts residents’ access to the internet [49,50]. Our study showed that compared with 18.93% (446/2355) of the urban residents using the internet, rural counterparts accounted for only 3.05% (179/5868). The policy implications of this finding highlight the importance of strengthening the network infrastructure in rural areas, such as the establishment of public Wi-Fi. In addition, friendly internet interfaces need to be designed to provide a better internet use experience for rural older adults.

### Limitations

Despite these important findings, this study has some limitations. First, the cross-sectional design limited our ability to draw any causal inference on the effect of internet use on urban-rural disparities in health outcomes. Second, the 3 dimensions of health outcomes were measured using self-report scales rather than clinical diagnostic measures. As a result, the health levels of the respondents may be over- or underreported. Third, the low internet use rate in this study, especially in rural areas, might underestimate the association between internet use and urban-rural disparities in health outcomes among older adults. Fourth, because of data availability limitations, only 2 variables related to internet use (probability and frequency of internet use) were used. Further research should consider adding other explanatory variables, such as variety, type, purpose, and duration of internet use. Fifth, the data may be old because the latest round of CHARLS was updated in 2018. However, our findings are still relevant for the following reasons. (1) The existence of a moderating effect of internet use on the association between urban-rural status and health outcomes is because the internet provides entertainment and communication functions for rural residents. In China, these functions are still widely used and effective for improving health. (2) The mediating effect of internet use on urban-rural disparities in health is explained by a digital divide between urban and rural areas. Owing to China’s dual urban-rural system, this digital gap is still large. In summary, the intrinsic factors driving the occurrence of moderating and mediating effects of internet use have not changed significantly, which makes our results applicable at present.

### Conclusions

Remarkable physical and psychological health disparities were observed between urban and rural older adults in China. These inequalities in psychological health are partially attributable to the urban-rural disparities in the prevalence of internet use. However, internet use is more conducive to improving the psychological health of rural users, thus eliminating rural-urban health inequalities in China. Strategies to reduce the urban-rural inequalities in mental health might be considered by providing convenient channels for the rural older adults to use the internet, improving the ability of rural users to effectively use the internet, and promoting internet popularity in rural areas.

## Appendix

Multimedia Appendix 1 Definition and measurements of study variables.

Multimedia Appendix 2 Full modifying effects models results.

## Abbreviations

CHARLS: China Health and Retirement Longitudinal Study
CSI-D: Community Screening Instrument for Dementia
KHB: Karlson-Holm-Breen
OR: odds ratio

## Data Availability

The data sets used and analyzed during the study are available on the internet [51].
