# Supplementary material for: Mediating and Moderating Effects of Internet Use on Urban-Rural Disparities in Health Among Older Adults: Nationally Representative Cross-Sectional Survey in China
Source: J Med Internet Res. 2023 Sep 28;25:e45343. doi: 10.2196/45343 (PMC10570902; doi:10.2196/45343)
Supplement: Multimedia Appendix 1 [file jmir_v25i1e45343_app1.docx]

**Multimedia Appendix 1**

**Table S1** **Definition and measurements of study variables**

| **Subgroup** | **Variable name** | **Type of variable** | **Measurements** |
| --- | --- | --- | --- |
| Outcomes | Functional disability | Categorical:  0=No  1=Yes | The basic activities of daily living (ADL) were used to assess self-reported needs for assistance in eating, dressing, indoor mobility, bathing, toileting, and continence. |
|  | Cognitive function | Categorical:  0=No  1=Yes | Cognitive assessment was conducted by using the brief Community Screening Instrument for Dementia (CSI-D). CSI-D consists of two parts: 1) an interview with the study participant measuring cognitive function and 2) an interview with a close relative to gather information on daily functioning and cognitive decline. The first part consists of seven cognitive test items including questions describing the use of a hammer, naming an elbow, pointing to the window and then to the door, locating the nearest store, orientation to season, orientation to day of the week, and delayed recall of three words. The second part consisted of six questions assessing changes in the respondent’s daily: worsening of the ability to speak, worsening of ability to think and understand, often forgetting where she/he had put things, always forgetting what happened the day before yesterday, sometimes not being able to recognize the current location, and difficulty in dressing. |
|  | Depression | Categorical:  0=No  1=Yes | Depressive symptoms were identified using the 10-item Center for Epidemiologic Studies Depression Scale (CESD-10). Respondents reported the frequency of occurrence of eight negative effect items and two positive effects items. |
| Independent variable | Residency | Categorical:  1=Rural  2=Urban | Current residential area of respondent. |
| Mediators/moderators | Internet use | Categorical:  0=No  1=Yes | Have you used the Internet in the past month? |
|  | Frequency of internet use | Categorical:  1=never  2=not regular  3=weekly  4=daily | How often in the last month used internet? |
| Control variables | Sex | Categorical:  1=Male  2=Female | Self-reported by respondents at the time of the survey. |
|  | Age | Continuous | Self-reported by respondents at the time of the survey. |
|  | Marital status | Categorical:  1=Married  2=Others | Current marital status reported by respondents at the time of the survey. |
|  | Literacy | Categorical:  0=No  1=Yes | Are you literate? |
|  | Wage/ pension/subsidy | Categorical:  0=No  1=Yes | Did you receive any wage/pension/subsidy in the past year? |
|  | Employment status | Categorical:  0=No  1=Yes | Did you engage in agricultural work or paid work in the past year? |
|  | Pension insurance | Categorical:  0=No  1=Yes | Do you currently receive, expect to receive, or contribute to the pension for public servants, or pension for public institution employees, basic pension for enterprise employees, supplementary pension insurance, Urban and Rural Resident Pension, New Rural Resident Pension, Urban Resident Pension, any commercial pension insurance or any other pension? |
|  | Medical insurance | Categorical:  0=No  1=Yes | Are you the policy holder/primary beneficiary of any of the types of health insurance listed below?  1. Urban employee medical insurance  2. Urban and rural resident medical insurance (integrated urban resident medical insurance and new rural cooperative medical insurance)  3. Urban resident medical insurance  4. New rural cooperative medical insurance  5. Government medical insurance  6. Medical aid  7. Private medical insurance: purchased by work unit  8. Private medical insurance: purchased by individual  9. Urban non-employed persons’ health insurance  10. Long-term care insurance  11. Other medical insurance |
|  | Access to physical examination service | Categorical:  0=No  1=Yes | Have you taken a physical examination in the past three years? |
