# Supplementary material for: Mediating and Moderating Effects of Internet Use on Urban-Rural Disparities in Health Among Older Adults: Nationally Representative Cross-Sectional Survey in China
Source: J Med Internet Res. 2023 Sep 28;25:e45343. doi: 10.2196/45343 (PMC10570902; doi:10.2196/45343)
Supplement: Multimedia Appendix 2 [file jmir_v25i1e45343_app2.docx]

**Multimedia Appendix 2**

**Table S1. Modifying effects of probability of Internet use**

|  | **Functional disability** | |  | **Cognitive function** | |  | **Depressive symptoms** | |
| --- | --- | --- | --- | --- | --- | --- | --- | --- |
|  | OR | 95%CI |  | OR | 95%CI |  | OR | 95%CI |
| **Residency (ref.=rural)** | |  |  |  |  |  |  |  |
| Urban | 0.826^b^ | (0.727, 0.939) |  | 0.473^c^ | (0.380, 0.588) |  | 0.802^c^ | (0.701, 0.918) |
| Internet use (ref.=no) | |  |  |  |  |  |  |  |
| Yes | 1.491 | (0.794, 2.799) |  | 0.052^c^ | (0.011, 0.260) |  | 0.620^a^ | (0.299,0.887) |
| **Gender (ref.= Male)** | |  |  |  |  |  |  |  |
| Female | 0.693^c^ | (0.629, 0.762) |  | 1.175^a^ | (1.024, 1.347) |  | 1.503 | (1.361, 1.661) |
| **Age** | 0.993 | (0.985, 1.001) |  | 1.059^c^ | (1.048, 1.071) |  | 0.999 | (0.991, 1.008) |
| **Marital status (ref.= married)** | |  |  |  |  |  |  |  |
| Others | 1.009 | (0.562, 1.811) |  | 0.411^b^ | (0.215, 0.787) |  | 0.917 | (0.508, 1.657) |
| **Literacy (ref.= no)** |  |  |  |  |  |  |  |  |
| Yes | 0.985 | (0.892, 1.088) |  | 0.296^c^ | (0.253, 0.346) |  | 0.780^c^ | (0.703, 0.865) |
| **Wage/ pension/subsidy (ref.= no)** | | |  |  |  |  |  |  |
| Yes | 1.040 | (0.923, 1.173) |  | 0.911 | (0.777, 1.067) |  | 0.894 | (0.790, 1.010) |
| **Employment status (ref.=no)** | |  |  |  |  |  |  |  |
| Yes | 0.956 | (0.863, 1.059) |  | 0.920 | (0.797, 1.061) |  | 0.839^b^ | (0.754, 0.939) |
| **Pension insurance (ref.=no)** | |  |  |  |  |  |  |  |
| Yes | 0.907 | (0.791, 1.040) |  | 0.357^b^ | (0.268, 0.475) |  | 0.627^b^ | (0.539, 0.729) |
| **Medical insurance (ref.=no)** | |  |  |  |  |  |  |  |
| Yes | 0.916 | (0.693, 1.210) |  | 0.786 | (0.564, 1.096) |  | 0.882 | (0.664, 1.170) |
| **Access to physical examination service (ref.=no)** | | | |  |  |  |  |  |
| Yes | 1.018 | (0.930, 1.114) |  | 0.725^c^ | (0.635, 0.825) |  | 0.897^a^ | (0.816, 0.986) |
| Rural-urban status* Internet use | 0.870 | (0.606, 1.248) |  | 7.327^c^ | (3.011,17.832) |  | 1.070^a^ | (1.037,1.787) |

^a^ *P*<.05, ^b^ *P*<.01, ^c^ *P*<.001

**Table S2. Modifying effects of frequency of Internet use**

|  | **Functional disability** | |  | **Cognitive function** | |  | **Depressive symptoms** | |
| --- | --- | --- | --- | --- | --- | --- | --- | --- |
|  | OR | 95%CI |  | OR | 95%CI |  | OR | 95%CI |
| **Residency (ref.=rural)** | |  |  |  |  |  |  |  |
| Urban | 0.827^c^ | (0.728, 0.940) |  | 0.471^c^ | (0.378, 0.587) |  | 0.799^c^ | (0.698, 0.914) |
| **Frequency of Internet use (ref.=never)** | |  |  |  |  |  |  |  |
| Not regular | 1.168 | (0.262, 5.207) |  | 5.545 | (0.668, 46.023) |  | 2.321 | (0.480, 11.225) |
| Weekly | 4.040 | (0.383, 42.611) |  | 0.515 | (0.029, 9.102) |  | 1.618 | (0.158, 16.587) |
| Daily | 1.409 | (0.696, 2.854) |  | 0.037^b^ | (0.005, 0.249) |  | 0.515^b^ | (0.223, 0.919) |
| **Gender (ref.= Male)** | |  |  |  |  |  |  |  |
| Female | 0.692^c^ | (0.629, 0.762) |  | 1.181^a^ | (1.028, 1.365) |  | 1.506^c^ | (1.363, 1.665) |
| **Age** | 0.993 | (0.985, 1.001) |  | 1.061^c^ | (1.049, 1.072) |  | 1.000 | (0.992, 1.008) |
| **Marital status (ref.= married)** | |  |  |  |  |  |  |  |
| Others | 0.859 | (0.483, 1.527) |  | 0.404^b^ | (0.211, 0.733) |  | 1.138 | (0.624, 2.074) |
| **Literacy (ref.= no)** |  |  |  |  |  |  |  |  |
| Yes | 0.985 | (0.892, 1.089) |  | 0.295^c^ | (0.252, 0.345) |  | 0.779^c^ | (0.703, 0.864) |
| **Wage/ pension/subsidy (ref.= no)** | | |  |  |  |  |  |  |
| Yes | 1.041 | (0.923, 1.174) |  | 0.944 | (0.804, 1.108) |  | 0.901 | (0.797, 1.019) |
| **Employment status (ref.=no)** | |  |  |  |  |  |  |  |
| Yes | 0.956 | (0.863, 1.059) |  | 0.919 | (0.796, 1.060) |  | 0.841^b^ | (0.756, 0.935) |
| **Pension insurance (ref.=no)** | |  |  |  |  |  |  |  |
| Yes | 0.909 | (0.792, 1.042) |  | 0.346^c^ | (0.260, 0.461) |  | 0.620^c^ | (0.533, 0.721) |
| **Medical insurance (ref.=no)** | |  |  |  |  |  |  |  |
| Yes | 0.916 | (0.693, 1.211) |  | 0.785 | (0.563, 1.095) |  | 0.882 | (0.664, 1.170) |
| **Access to physical examination service (ref.=no)** | | | |  |  |  |  |  |
| Yes | 1.017 | (0.929, 1.113) |  | 0.724^c^ | (0.636, 0.824) |  | 0.895^a^ | (0.814, 0.983) |
| Rural-urban status* not regular | 0.951 | (0.346, 2.613) |  | 0.573 | (0.105, 3.122) |  | 0.631 | (0.210, 1.900) |
| Rural-urban status* weekly | 0.421 | (0.112, 1.591) |  | 4.061 | (0.762, 21.661) |  | 0.904 | (0.241, 3.401) |
| Rural-urban status* daily | 0.911 | (0.610, 1.360) |  | 8.580^c^ | (3.018, 24.392) |  | 1.190^b^ | (1.037, 1.923) |

^a^ *P*<.05, ^b^ *P*<.01, ^c^ *P*<.001
